# Identification of Potential Binding Sites of Sialic Acids on the RBD Domain of SARS-CoV-2 Spike Protein

**DOI:** 10.3389/fchem.2021.659764

**Published:** 2021-07-22

**Authors:** Bingqian Li, Lin Wang, Huan Ge, Xianglei Zhang, Penxuan Ren, Yu Guo, Wuyan Chen, Jie Li, Wei Zhu, Wenzhang Chen, Lili Zhu, Fang Bai

**Affiliations:** ^1^ Shanghai Institute for Advanced Immunochemical Studies and School of Life Science and Technology, ShanghaiTech University, Shanghai, China; ^2^ Department of Chemistry, Imperial College London, London, United Kingdom; ^3^ State Key Laboratory of Bioreactor Engineering, Shanghai Key Laboratory of New Drug Design, School of Pharmacy, East China University of Science and Technology, Shanghai, China; ^4^ State Key Laboratory of Medicinal Chemical Biology and College of Pharmacy, Nankai University, Tianjin, China; ^5^ National Center for Protein Science Shanghai, Shanghai, China

**Keywords:** SARS-CoV-2, receptor, sialic acid, RBD domain, spike protein

## Abstract

COVID-19, caused by severe acute respiratory syndrome coronavirus 2 (SARS-CoV-2), is still an emergent pandemic for humans. The virus infection is achieved by penetrating its spike protein to host cells via binding with ACE2. Moreover, recent studies show that SARS-CoV-2 may have multiple receptors that need to be further revealed. SARS-CoV-2 shares similar sequences of the spike protein with the Middle East Respiratory Syndrome Coronavirus (MERS-CoV), which can invade host cells by binding to either DPP4 or sialic acids. Sialic acids can be linked to the terminal of glycoproteins and gangliosides are used as one of the receptors of many types of viruses. Therefore, it is very interesting to determine whether sialic acid is a potential receptor of SARS-CoV-2. To address this question, we took N-Acetylneuraminic acid (Neu5Ac), a type of predominant sialic acid found in human cells, as the molecular probe to computationally search the surface of the spike protein to locate the potential binding sites of Neu5Ac. SPR analysis and mass spectrum analysis confirmed the interaction between Neu5Ac and spike protein. This study shows that sialic acids can moderately interact with the spike protein of SARS-CoV-2 by binding between the two RBDs of the spike protein, indicating it could be a potential secondary or auxiliary receptor of SARS-CoV-2.

## Introduction

The new coronavirus disease 2019 (COVID-19) is caused by severe acute respiratory syndrome coronavirus 2 (SARS-CoV-2). COVID-19 has caused a worldwide health emergency with parallel effects on the economy. Over a hundred million cases were reported by February 20, 2021, with thousands of deaths every day (World Health Organization, 2021). The molecular mechanisms of SARS-CoV-2 infection are still not clear and urgently needed to be explored. To date, several medical agents, including small molecular agents and vaccines are in the process of clinical trials (Liu et al., 2020).

SARS-CoV-2 belongs to the beta-coronavirus family which contains Human beta-Coronavirus (HCoV-OC43), Human beta-Coronavirus (HCoV-HKU1), Severe Acute Respiratory Syndrome Coronavirus (SARS-CoV), and the Middle East Respiratory Syndrome Coronavirus (MERS-CoV) (Hu et al., 2015; Hulswit et al., 2019). It shares similarity in sequence with SARS-CoV and MERS-CoV, being with identity of 79 and 50%, respectively, (Lu et al., 2020; Petrosillo et al., 2020) Compared with MERS-CoV and SARS-CoV, the SARS-CoV-2 virus has a relatively low mortality rate (around 2.3%) (9.5% for SARS-CoV and 34.4% for MERS-CoV), but a significantly higher rate of transmission (DÖmling and Gao, 2020; Petrosillo et al., 2020).

Coronavirus enters the host cell mainly by binding to the host cell receptor. Both SARS-CoV and SARS-CoV-2 share the same human cell receptor, angiotensin-converting enzyme 2 (hACE2), while MERS-CoV enters human cells by binding to dipeptidyl-peptidase (DPP4) (Wan et al., 2020). Cell surface protease TMPRSS2 and lysosomal cathepsins activate the SARS-CoV-2 and may cleave the spike protein at two distinct sites. This presence of pre-activation enables SARS-CoV-2 to be less dependent on target cell activation. Studies have also shown a higher binding affinity to *h*ACE2 for SARS-CoV-2 than for SARS-CoV (Hoffmann et al., 2020). Two other potential host receptors for SARS-CoV-2 entry, kringle containing transmembrane protein 1 (KREMEN1) and asialoglycoprotein receptor 1 (ASGR1), were recently discovered (Gu et al., 2020).

Sialic acid is a generic term for a family of derivatives of neuraminic acid, an acid sugar with a nine-carbon backbone. It is generally found in the terminal position on a variety of glycoconjugates, which cover the surfaces of many different cell types, playing important cellular functions, including mediating the attachment, and entry of types of viruses, such as influenza viruses, orthomyxoviruses, infectious salmon anemia virus, as well as coronavirus (Matrosovich et al., 2015). HCoV-OC43 and HCoV-HKU1 can interact with 9-O-acetyl-sialic acid to infect the host cell (Hulswit et al., 2019; Tortorici et al., 2019). Different from HCoV-OC43, MERS-CoV also shows a stronger preference interaction with α2,3-linked sialosides other than α2,6-linked sialosides (Park et al., 2019). One recent study reported the identification of binding between SARS-CoV-2 and sialic acids (N-acetyl neuraminic acid) by using a new lateral flow detection system. (Baker et al., 2020) This indicates that sialic acids may be a candidate receptor, and their binding molecular mechanisms with spike protein need to be further studied.

SARS-CoV-2 is formed as an enveloped structure that contains RNA genome, spike (S) protein, nucleocapsid (N) protein, membrane (M) protein, and envelop (E) protein. The homo-trimeric S protein contains two subunits, S1 and S2, covering the cleavage sites at R685 and S686 (Hu et al., 2015; Woo et al., 2020). The N-terminal S1 subunit mainly comprises the N-terminal domain (NTD) and receptor-binding domain (RBD), which is responsible for *h*ACE2 binding. However, KREMEN1 and ASGR1 bind to both NTD and RBD (Gu et al., 2020). The C-terminal S2 subunit is mainly made up of heptad repeats 1 and 2 (HR1 and HR2), as well as the transmembrane domain (TM), which specializes in membrane fusion while entering the cell (DÖmling and Gao, 2020; Woo, et al., 2020).

The spike glycoprotein of SARS-CoV-2 is usually in a “down” conformational state to escape from the immune response. When it approaches a target cell receptor, RBD shifts its position to bind with a human cell receptor, which turns the protein into an “up” conformational state (Shang et al., 2020). The types of conformational structures of S protein and compositions were fully discovered using cryo-EM, with 31% S protein in the “down” conformational state, 55% in the state with one RBD “up”, and 14% in the state with two RBDs “up” (Cai et al., 2020; Ke et al., 2020). In reality, S protein is largely shielded by glycans, which are utilized for thwarting immune response from the host. N*-*glycans at N165 and N234 play a critical role in the process of the state changes of RBD (Casalino et al., 2020) Previous studies have predicted 22 N-glycosylation and 4 O*-*glycosylation sites on the surface of S protein (Woo et al., 2020). 17 of 22 N-glycosylated and 2 O-glycosylated sites were observed using the cryo-EM technique. (Shajahan et al., 2020; Woo et al., 2020).

Computational techniques have already been widely used in drug discovery. Although experimental technologies provide straightforward observation in studies, they are normally time-consuming and laborious. Moreover, recent techniques focus on studying biological molecular mechanisms by using molecular dynamics (MD), which improve the understanding of reaction mechanisms and protein dynamic behavior (Karplus and McCammon, 2002). For example, Arantes’ group used MD simulations to explore strategies for developing vaccines of SARS-CoV-2 (Arantes et al., 2020). Deganutti’s group focused on identifying druggable binding sites on the SARS-CoV-2 spike protein by using supervised molecular dynamics. (Deganutti et al., 2021). Chauhan’s team outlined some key aspects in molecular structure that may affect inhibition performance in organic corrosion inhibitors using molecular dynamics techniques (Chauhan et al., 2021).

Yadav’s group tested FDA-approved drugs on several new SARS-CoV-2 proteases using molecular docking techniques (Yadav et al., 2020).

The present study designed a comprehensive framework by combining multiple computational modeling methods with experimental technologies, aiming to determine whether and how sialic acids bind with the spike protein of SARS-CoV-2. Several studies have shown glycosylation can alter the thermodynamic stability and folding as well as conformations of proteins, resulting in an increase in protein free energies (Shental-Bechor and Levy., 2008; Gavrilov et al., 2015). Hence, this work also studies whether the binding of sialic acids may also be affected by the existence of glycan ligands on the surface of the spike protein.

## Results and Discussion

### Identification of Possible Binding Sites of Sialic Acids

To explore the potential binding sites of sialic acids on the surface of the spike protein, in which both the conformational change and the glycosylation states were considered, a series of ligandable binding site identification simulations were performed on the four different modeled protein structures of the spike protein (illustrated in Supplementary Figure S1). Both RBD “down” and one RBD “up” conformations, as well as the glycosylation, were considered. As a result, four protein structures were constructed based on the different RBD conformational states and glycosylated states. A detailed description of the protein structure modeling process has been given in the methods section. Firstly, twenty-one potential binding sites mainly locating on the S1 domain were identified by using Sitemap (Halgren, 2009), which is a computational druggable binding site characterizing method for proteins. To double-check the prediction results, another binding site identification method, which was a fragment-based druggable “hot spot” searching method developed with a different algorithm (the Fourier domain correlation algorithm), named as FTMap, was also used to search again on the surface of these four structures. The spike protein is trimer and some positions identified should be symmetrically located. However, some of such binding sites were only captured once or twice in our calculations. In this way, we artificially corrected the results by adding the missing ones. Finally, forty candidate positions were obtained. Interestingly, the obtained active sites were shown at similar positions with the results from Sitemap. These candidate binding sites should be evaluated further by using other techniques.

**FIGURE 1:**
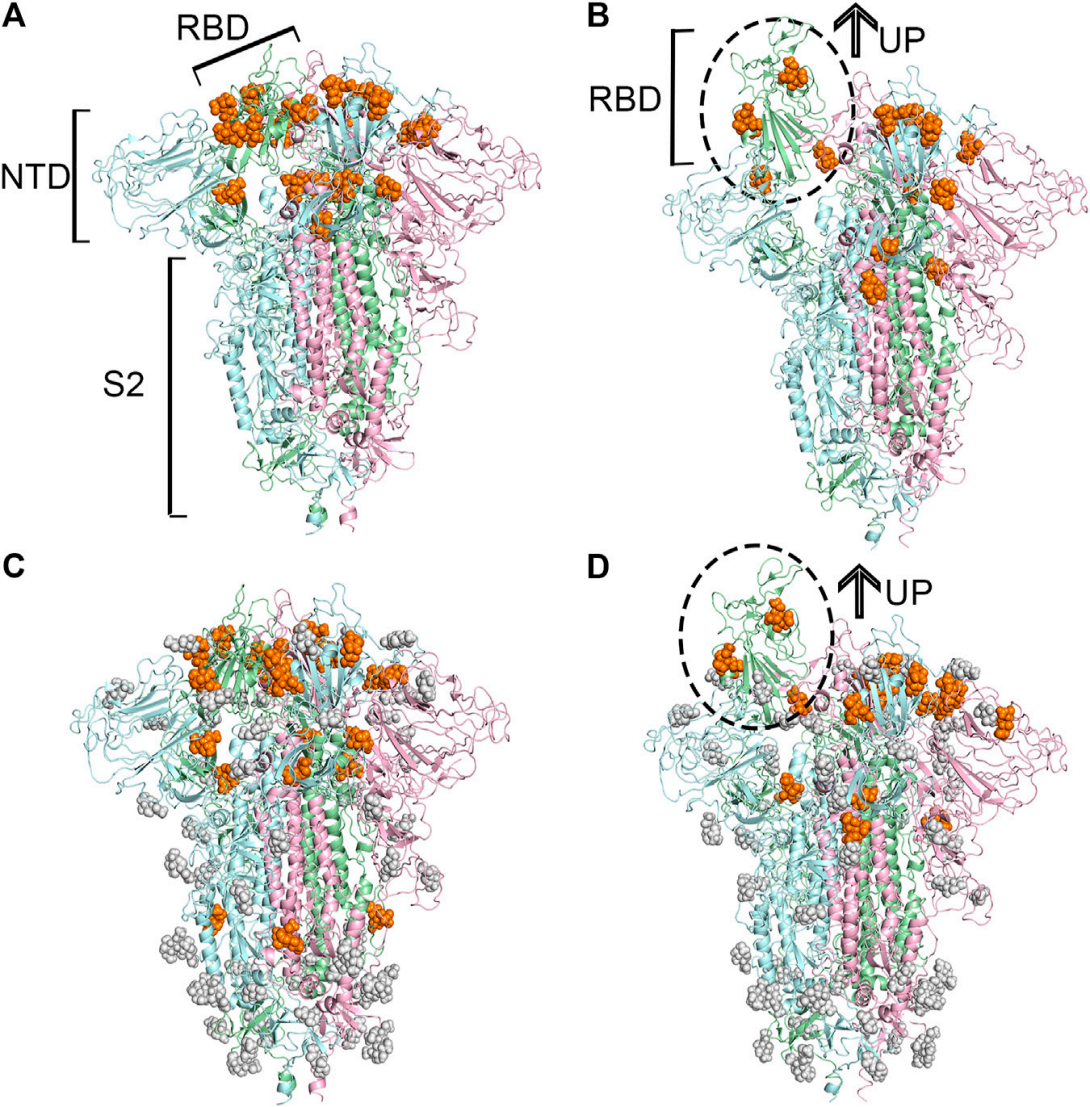
Predicted potential bindings of sialic acids on the surface of the spike protein. Potential ligandable binding sites were identified on the four constructed models of the trimeric spike protein by using FTMap (Ngan et al., 2012) and SiteMap (Halgren, 2009). The different colors of the cartoon models in each figure represent different chains of the protein: chain A is shown in pale-green, chain B is in pale-blue, and chain C is in light-pink. The protein structures which contain gray sphere balls represent glycosylated state S trimer (the gray sphere models are the glycosylation), and the orange balls represent sialic acid molecules. **(A)** 21 and **(B)** 15 sialic acid molecules were observed to bind to the different places of the surface of unglycosylated spike protein in “down” and “up” conformational states, respectively, **(C)** 23 potential sites for sialic acid binding were identified on the surface of glycosylated spike protein in “down” conformational state, and 17 were found **(D)** in the “up” conformational state.

Based on these identified candidate binding sites, several rounds of molecular docking simulations were performed to verify whether sialic acids could interact with or not. Theoretically, the sialic acid may be extended with oligosaccharides to decorate glycoproteins and gangliosides at the host cell surface (Schauer and Kamerling, 2018). Hence, the binding sites of sialic acids could be relatively exposed to solvent, in other words, on the surface of a protein. Given this, the candidate binding sites located on the surface of proteins were extensively explored. Because of this, we used N-Acetylneuraminic acid (Neu5Ac), a type of predominant sialic acid, as a small molecular probe to detect the potential anchor site of glycogen chains on the surface of the S protein. The spike protein is a pivotal trimeric structure, therefore, once the candidate binding sites were discovered on one chain of the protein, the additional binding sites symmetrically located on the other chains would be manually added to our candidate list if they were not observed accidentally. Finally, for the protein in the “up” conformational state, 15 potential binding sites on the unglycosylated spike protein, and 17 on the glycosylated spike protein were observed, respectively. Compared with the “up” state, 21 and 23 potential binding sites were found to be able to accommodate sialic acids in the unglycosylated and glycosylated “down” state of the protein. Through the comprehensive analysis of the locations of all the candidate binding sites, a total of 40 unique candidate binding sites were collected from those four protein models and numbered from 1 to 40. In general, most of the predicted binding sites of sialic acid molecules were found to locate in the RBD of the protein, as shown in Figure 1. Based on the above docking simulations and artificial correlation, these four modeled protein structures were modeled as multiple sialic acid-bound complex structures.

### Determining the Most Likely Binding Site for the Sialic Acid

**FIGURE 2:**
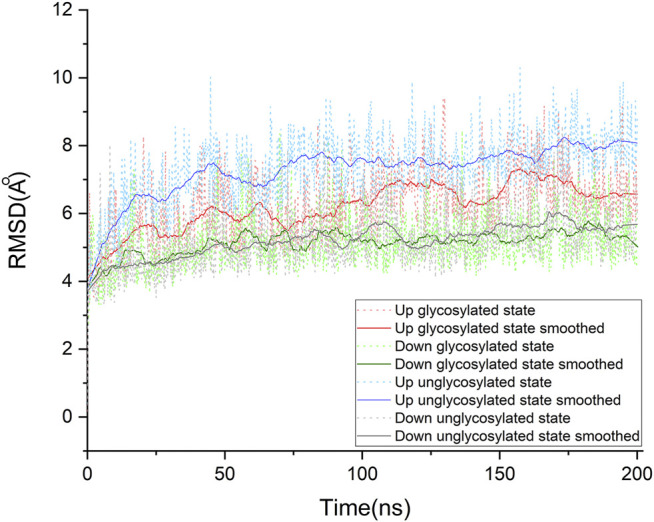
Cα root-mean-square deviation (RMSD) of the molecular dynamics simulations for our four systems. Each system was studied extensively by running three times of molecular dynamic simulations. For each protein system, only one of the simulations was taken out to make this plot representative. The raw data of RMSDs are shown in dot lines, and the fluctuations of RMSD are smoothed by using the Savitzky-Golay method in OriginPro, version 2020 (OriginLab Corporation, Northampton, MA, and United States), with the polynomial order as 1 and polynomial order as 50. The details for other trajectories are shown in supporting information (See *S*upporting Information. Supplementary Figure S2). All systems show an RMSD variation around 3 ∼ 4 Å, which is contributed by the large conformational motion of flexible loops of the spike protein. Compared with these “down” conformational states, these “up” conformational states show more obvious fluctuations.

**FIGURE 3:**
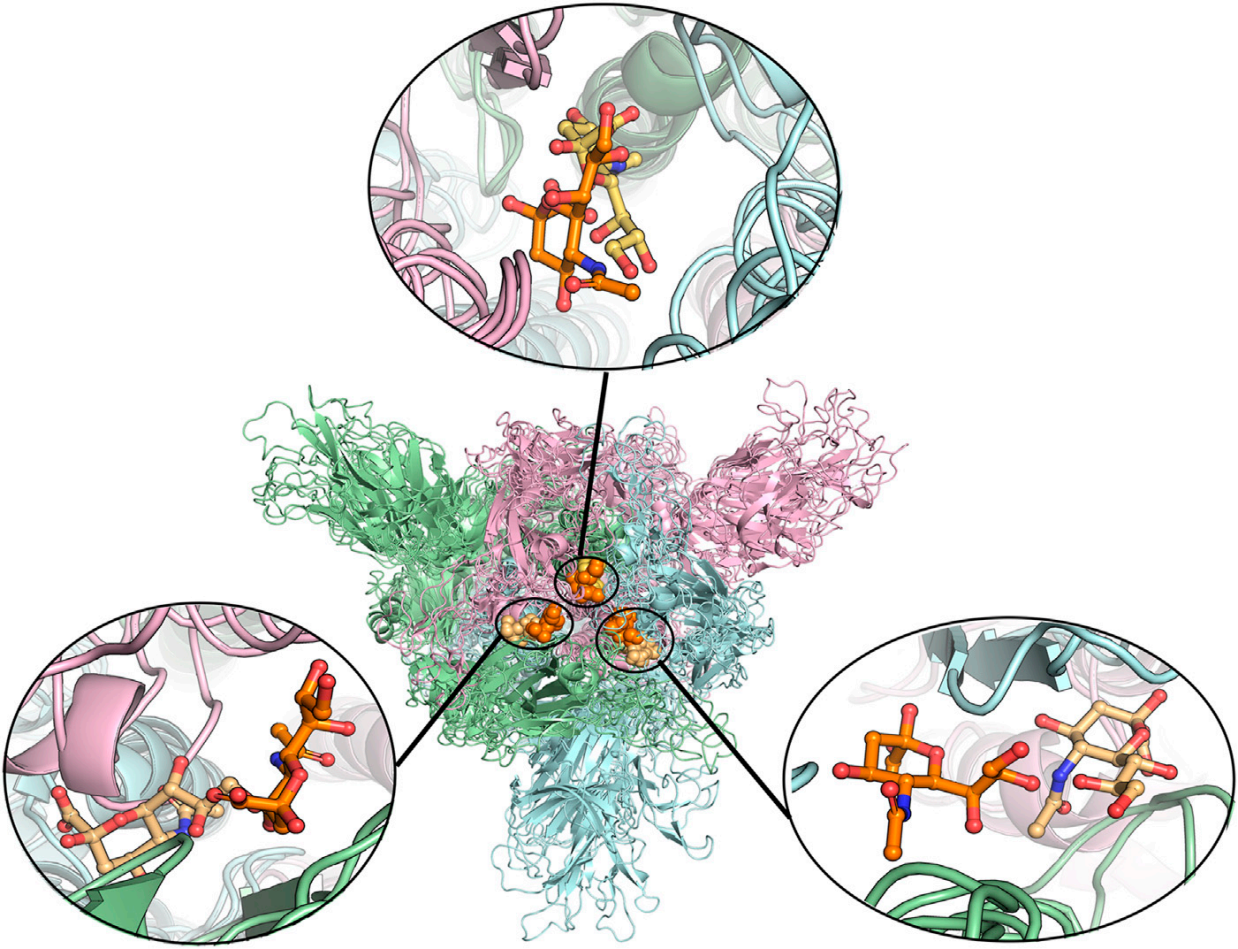
Alignment of different protein structures to identify the overlapped sialic acid-binding positions, that is, SA_6, SA_7, and SA_24. The figure shows the alignment of these final stable complex structures of sialic acid with spike protein, generated by the MD simulations starting from those four different protein structures. The sialic acid-binding positions, i.e., SA_6, SA_7, and SA_24 were found to be conserved in “down” conformational states of the spike protein and to be observable in “up” conformational states. Position SA_6 is between chain B (pale-blue) and chain C (light-pink), position SA_7 is between chain A (pale-green) and chain B and position SA_24 is between chain A and chain C. The stable bound sialic acids are shown in different colors. Three sialic acids in the glycosylated “down” conformational state are shown in orange color. Two sialic acids in the unglycosylated “down” conformational state are shown in light orange color. One sialic acid bound on the glycosylated “up” conformational state is shown in yellow-orange color. The detailed interaction modes for the bound modes are shown in Figure 4 and Supplementary Figures S7, S8. No stable sialic acid appears in these three positions for the glycated “up” state.

To find the most likely binding site for the sialic acid, where a sialic acid molecule could stably bind, a series of molecular dynamics simulations were carried out to monitor the stability of the bindings of sialic acids against the candidate binding sites, obtained from the molecular docking simulations mentioned above. Theoretically, the weaker bound sialic acids would dissociate faster. For each protein structure, three repeated MD simulations were performed. These modeled four sialic acid-protein complex structures were subjected to molecular dynamics simulations for 200 ns and generated twelve independent trajectories. As shown in Figure 1. The Cα-RMSD of each trajectory shows relative fluctuations of the proteins in a range of about 3–4 Å (Figure 2; Supplementary Figure S2). These curves which show obvious fluctuations of the conformational change of the protein along the trajectories are mainly contributed by the larger numbers of flexible loops of the protein. The “down” states of S protein generally show relative small-scale fluctuations than “up” states, suggesting that later structures could be less stable. During the simulations, some sialic acid molecules docked to the protein surface fly away after 20 ns of simulations, whereas some are stably staying in their positions after 200 ns. Therefore, the most probable (strongly bound) sites for the sialic acid can be distinguished from others. To compare the strength of those binding sites, all unique potential pockets were numbered from 1 to 40 by simply aligning all four structures. The strength of interactions of every ligand (sialic acid) in each frame of the trajectories was analyzed and plotted in heatmaps (See Supporting Information, Supplementary Figures S3–S6). Moreover, the depth of color suggests the number of molecular interactions, i.e., hydrogen bonds, hydrophobic contacts, and ionic bonds, etc., between ligand and protein in each frame. Interestingly, we found some sialic acids that flew away after 20 ns and re-bounded to the protein at different positions from their initial binding sites, which then left again after several nanoseconds. However, this action did not show up in any repeats for a particular sialic acid and therefore is random interactions. From an overall perspective, the glycosylation may be beneficial to strengthening the binding of sialic acids to the spike protein, as the interactions observed in the glycosylated spike protein are significantly more than unglycosylated proteins (as shown in Supplementary Figures S3–S6). By comparing the heatmaps, sialic acids at positions SA_6, SA_7, and SA_24 are appeared to show stable interactions within the 200 ns trajectories for glycosylated “RBD” down conformational state (Supplementary Figure S3). The positions of SA_7 and SA_24 can also be observed to be stable within the 200 ns trajectories for unglycosylated “RBD” down conformational state (Supplementary Figure S4). Sialic acid is strongly bound to the position of SA_6 in glycosylated “RBD” up conformational state (Supplementary Figure S5). By superposing the last frames from the trajectories for these four protein structures (Figure 3), we found that the positions of SA_6, SA_7, and SA_24 are similar and symmetrically located between every two adjacent RBDs from different chains.

**FIGURE 4:**
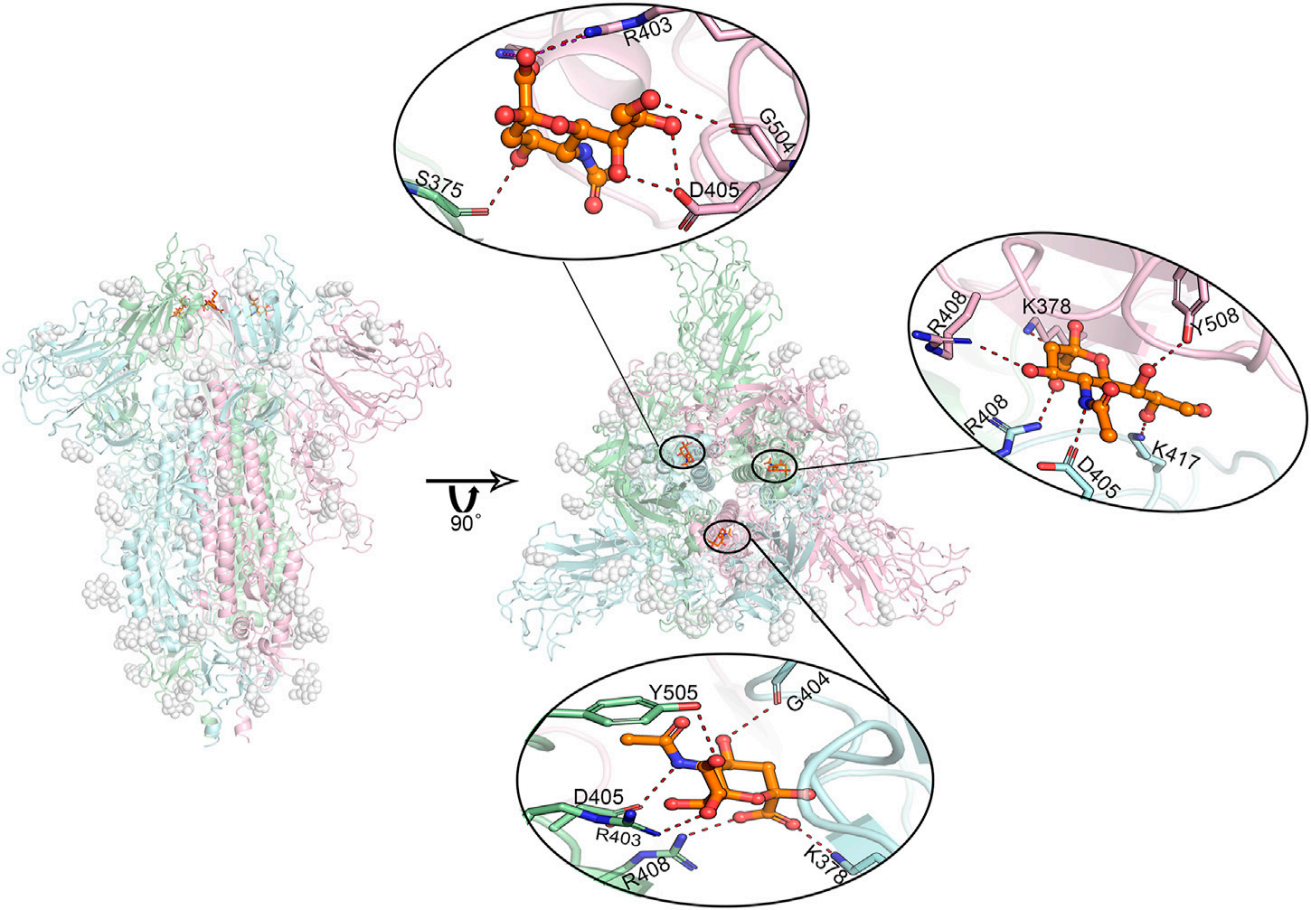
Predicted most likely binding modes of sialic acids with glycosylated spike protein at positions SA_6, SA_7, and SA_24. Molecular dynamics simulations identified three stable interacting sites, locating between every two adjacent RBD domains. The figure in the middle shows the relative positions of these three bound sialic acids. The enlarged binding areas show the detailed molecular interactions at each binding site. Position SA_6 is between chain B (pale-cyan) and chain C (light-pink). Sialic acid can form hydrogen bonds with the residues of R403, G504 as well as D405 from chain B, and the residue of S375 from chain C. Position SA_7 locates between chain A (pale-green) and chain B, where the sialic acid molecule is observed to specifically interact with the residues of R403, D405, R408 and Y505 of chain A, and the residues of K378 as well as G404 of chain B in the form of salt bridges or hydrogen bonds. Position SA_24 lies between chain A and chain C, where the sialic acid binds to the residues of D405, R408 as K417 of chain B, and the residues of K378, R408, and Y508 of chain C. Overall, the residue D405 in each of these three chains shows a significant role in sialic acid binding. The interactions to K378/S375 on their adjacent chains may play a role in further stabilizing sialic acid molecules. Dash lines represent hydrogen or ionic bonds. White spheres are the glycans that are artificially modified on the protein.

The predicted binding modes of sialic acid molecules in the positions of SA_6, SA_7, and SA_24 for glycosylated “RBD” down conformational state are shown in Figure 4. In general, the residue of D405 cooperated with its neighboring residue of R403 or R408, participating in all sialic acid interactions in the positions of SA_6, SA_7, and SA_24. On the other hand, sialic acids form a salt bridge with K378 of an adjacent chain, whereas in the position of SA_24, the predicted binding orientation is slightly different from the other two positions, i.e., interacting with the residue of S375 but not K378. Three sialic acid molecules symmetrically bind around the residue of D405 in each chain with forming couples of molecular interactions, such as the hydrogen bonds with G504, G404, and K417. Moreover, the observed binding mode of sialic acid molecules against these three positions in the different conformational or glycosylation states are shown in Supplementary Figures S7, S8. Both “down” state systems show sialic acids stably bind between two adjacent RBDs in different chains, whereas in the “up” state system, there is only one sialic acid in the same position because one “up” chain of RBD could distort the binding sites (Supplementary Figure S7). Alternatively, this can be explained by the shifting position of one RBD. As the RBD shifting upwards, it moves further from the other two RBDs, causing loss of stable interactions. The positions of sialic acids in each trajectory are slightly shifted from the origin docking position but still in the same region. These findings indicate that sialic acids could bind to the RBD domain of the spike protein of SARS-CoV-2, but not the N-terminal domain of the S1 domain (NTD) that the binding sites of the sialic acid locate on the MERS’s or other viruses’ spike protein. Therefore, experimental validations were performed against Neu5Ac and the RBD of the spike protein.

On the other hand, as shown in Supplementary Figures S3–S6, SA_28 shows an obvious preference for binding with sialic acid. The binding mode has been illustrated in Supplementary Figure S9. This position is embedded inside of the protein. Despite its strong interaction with sialic acid, it should not be the binding site for glycans.

One recent study reported conformational accessibility and binding strength of the S protein to its receptor of ACE2. In these reported simulations, five potential ligand-binding pockets were identified to expose and correlate with the conformational shifts of S protein (Peng et al., 2020). The authors also screened the compound database to identify potential ligands and reported one polyhydroxy (Quercetin) compound that is somehow like the sialic acid. This makes us curious whether this pocket is the site of the sialic acid. By carefully comparing, pocket four was found in the report to be close to our predicted site, but not fully overlapping. This indicates that the binding of the sialic site in this position may be involved in some relationship with the conformational change of spike protein, but we do not know how and why at this stage. It is a very interesting topic that needs to be explored further in the future.

### Mass Spectrometry Analysis

**FIGURE 5:**
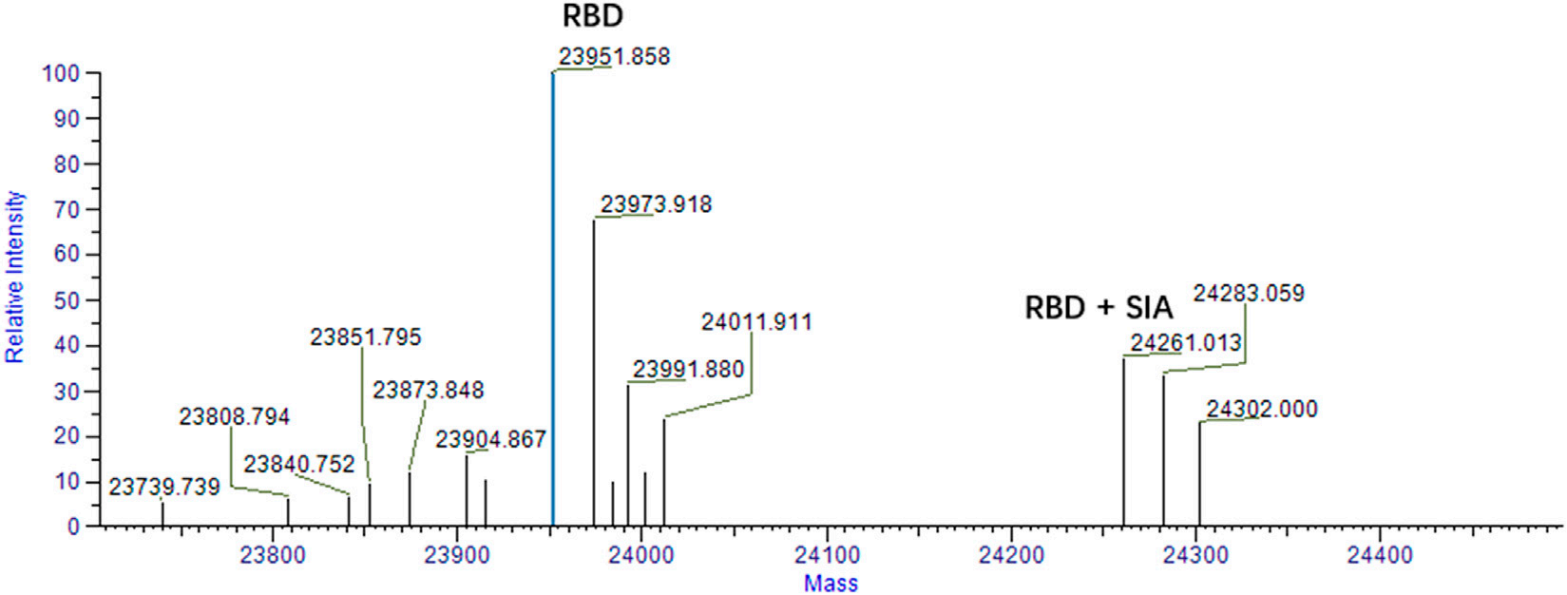
Mass spectrum analysis of RBD and sialic acid binding complex. The peak at 23,951 represents the RBD domain. The ligand-protein binding complex is shown by the peak 24261 m/z. The condition for analysis is RBD: SIA = 1:50.

To validate the binding of sialic acid to spike protein. Mass spectrometry analysis was firstly performed to qualitatively determine the bindings. According to our computational prediction, sialic acids may bind the site which is around the residue of D405 and belongs to the RBD domain. Therefore, mass spectrometry analysis was carried out between sialic acids and the RBD domain of spike protein. The experiment was carried out in a protein-ligand ratio of 1:50. As shown in Figure 5, the presence of a peak at 24,261 suggests the presence of a ligand-protein complex. The ratio of peaks at 23,951 and 24,261 is approximately 2.5:1.

### Surface Plasmon Resonance Analysis

**FIGURE 6:**
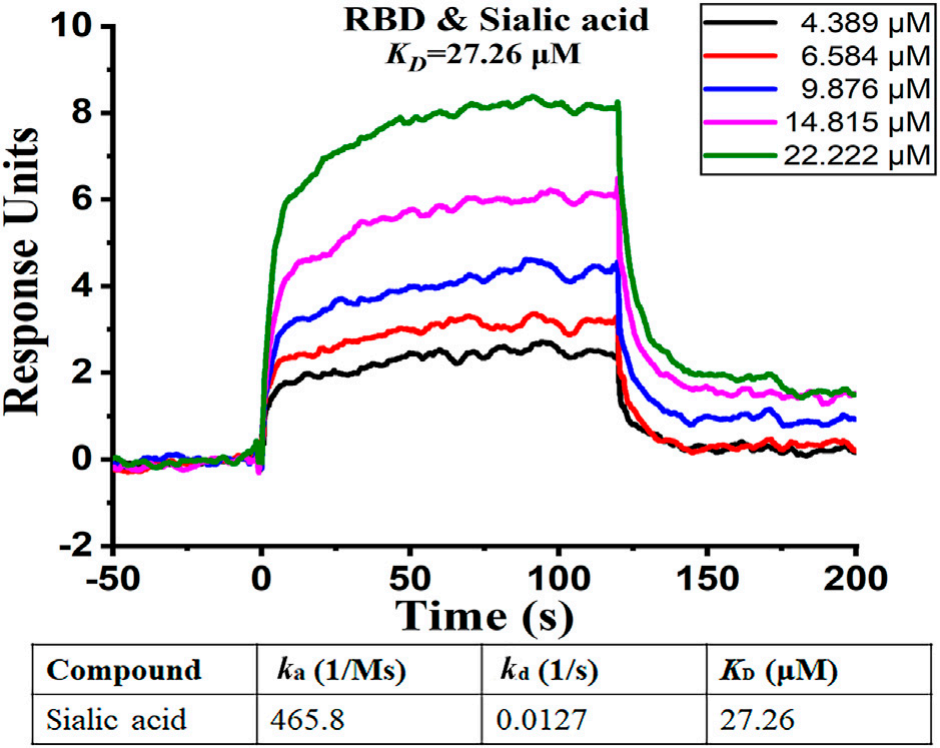
SPR analysis of binding affinity between sialic acid and RBD domain. The figure shows different levels of response with different sialic acid concentrations. The curve suggests the presence of binding between sialic acid and the RBD domain.

To further confirm our findings, we analyzed the binding affinity between the RBD domain and sialic acid by using Surface Plasmon Resonance (SPR). SPR is a biophysical method that can quantify molecular-molecular binding interactions. It allows “label-free” detection in real time and has been widely used to monitor interface processes (Shepherd et al., 2014). The experimental result shows that sialic acid effectively binds to the RBD domain with a rapid dissociation rate (*k*_off_ = 0.0127 1/s), and the binding is concentration-dependent, as illustrated in Figure 6. The experiment shows a binding affinity of *K*_D_ = 27.26 μM. From previous computational simulations, sialic acid is most likely bound between two adjacent RBD domains, while the SPR only detects the interaction between ligand and one RBD (1:1 model). In other words, the real binding affinity should be stronger than the observed 27.26 μM.

### Further Discussion

SARS-CoV-2 is experiencing rapid evolution. A number of mutations have been observed and most of them have occurred on the spike protein (CNCB, 2021; CoVariants, 2021). At present, the most important reported mutations are D614G and N501Y, which have been found can increase the binding affinities between the spike protein and ACE2. The two mutations are away from the identified bindings sites of sialic acid, and may not impact their bindings (Leung et al., 2021; Weissman et al., 2021). We also compared all reported single point mutations of S protein of SARS-CoV-2 (Supplementary Table S1), and mutations that happened outside the proposed binding sites for sialic acids, therefore, are less likely to affect the binding of sialic acids.

This paper proposes a new binding site for sialic acids on the RBD domain of Spike proteins. Drug repurposing can be done on this pocket through artificial intelligence (Zhou et al., 2020). Apart from Yadav’s work, last year, Martin’s group reported that Toremifene, an FDA-approved drug, could work on SARS-CoV-2 S protein and NSP14 (Martin and Cheng., 2020). This provides a new scientific orientation for further studies.

Moreover, the allosteric binding sites on the S protein of SARS-CoV-2 have been discovered and reported by designing a comprehensive framework, combining computational methods and experimental validation (Paola et al., 2020). The allosteric sites, being different from the conventional active sites, can allosterically alter the conformation of the proteins and regulate the functions. Therefore, it is important to identify the potential allosteric binding sites of sialic acids and probe the corresponding allosteric molecular mechanisms, to better understand the functions of sialic acids in triggering the virus invasion.

## Methods

### Constructing the Spike Protein Structures

Full-length spike protein structure models were built based on experimentally obtained protein structures, the PDB codes of these proteins are 6VXX for “down” and 6VSB for “up” conformational states. The missing fragments of the sequence were added by comparing different spike protein PDB models. Gaps between loops were filled by referring to full-length sequence of S protein by using Maestro (Zhu et al., 2014). A part of the incomplete RBD in all three chains was replaced by a modeled fully-sequenced model (modification based on the structure with the PDB code of 6M17). Based on the built-up reference models, two spike protein trimer models, one “up” and one “down” conformational state, was built using the Maestro Homology modeling method (Cappel et al., 2016). The glycosylation in the above PDBs were kept, and an additional one missing O*-*glycosylation at N801 was added manually according to the literature (Woo et al., 2020). The other two models without glycans were built by removing the glycans from previously built structures (Supplementary Figure S1)

### Detecting Potential Druggable Binding Sites for Sialic Acids on Spike Protein

Based on the above built four protein structures, SiteMap (Halgren, 2007; Halgren, 2009) and FTMap (Ngan et al., 2012) were used as two individual methods, which gave complementary results, identifying active sites on the surface of the spike protein structure. The SiteMap is a server of Schrödinger which predicts possible binding sites by scanning through the protein surface (Halgren, 2007). FTMap scans the entire protein by placing lots of probes in the funnel. More detailed descriptions of FTMap have already been published (Brenke et al., 2009; Ngan et al., 2012; Kozakov et al., 2015). Both methods were used by setting the parameters as default. Compared with SiteMap, the binding sites found by FTMap were relatively embedded into the protein. Overall, approximately 21 candidate sites were detected on the surface of the trimer. Then, sialic acid molecules were placed onto the trimer structure using molecular docking simulations by Glide (Tubert-Brohman et al., 2013). Two potential binding sites on “down” glycosylated state were added artificially because there were similar binding sites had been observed on the symmetric chain.

### Molecular Dynamics Simulations

All simulation systems were built and minimized using Desmond (Bowers et al., 2006), TIP3P (Gillan et al., 2016) as water model, neutralized by Na cation and Cl anion. Simulations for “down” state without glycans initially measured 45,770,357 Å^3^, NaCl at a concentration of 0.15 M, for a total of about 440,000 atoms. Simulation for “down” state with glycans initially measured 4,804,764 Å^3^, NaCl at a concentration of 0.15 M, for a total of about 442,000 atoms. Simulation for “up” state without glycans initially measured 4,770,069 Å^3^, NaCl at a concentration of 0.15 M, for a total of about 447,000 atoms. Simulation for “down” state with glycans initially measured 4,902,837 Å^3^, NaCl at a concentration of 0.15 M, for a total of about 460,000 atoms. The systems were modeled in the OPLS_2005 force field (Shivakumar et al., 2010). A molecular dynamics simulation was carried out using Desmond (Bowers et al., 2006). The systems were pre-production run for 50 ns After that, each trajectory was set for a longer simulation as long as 200 ns, an ensemble at 310 K (37°C), and 1 bar. Every system was then repeated three times with the same conditions but various initial velocities. Trajectories were analyzed using a simulation interaction analysis module in Maestro (Bowers et al., 2006).

### Mass Spectrometry Analysis

Proteins were dissolved in 25 ammonium acetate at a concentration of 10 uM, the drugs dissolved DMSO were diluted by 25 ammonium acetate to 100 uM. Then proteins were incubated with an equal volume of the drugs.

The above-mixed solutions were then injected into Orbitrap Fusion MS (Thermo Scientific) through direct injection. The MS was operated in intact protein mode. Data were analyzed with BioPharma Finder (Thermo Scientific) software (Marcoux et al., 2015).

### Surface Plasmon Resonance Analysis

We carried out surface plasmon resonance (SPR) experiments using BIAcore T200 to evaluate the kinetic parameters of sialic acid binding to RBD. The purified RBD (residues 319–591), which was diluted in sodium acetate solution (pH 4.5) with a final concentration of 50 μg/ml, was immobilized covalently on a CM5 sensor chip. The final immobilization level was 4,430.3 resonance units (RU). The running buffer was PBS, 0.005% (vol/vol) surfactant P20, pH 7.4, and 1% DMSO. Salic acid was diluted using the running buffer from the top concentration. The measurements were performed at a flow rate of 30 μL/min. For each binding cycle, the analyte was injected for 120 s and the dissociation time was 180 s. Data were analyzed using BIAevaluation 1.1 software.

## Data Availability

The raw data supporting the conclusions of this article will be made available by the authors, without undue reservation.
