# Supplementary material for: Identification of Potential Binding Sites of Sialic Acids on the RBD Domain of SARS-CoV-2 Spike Protein
Source: Front Chem. 2021 Jul 22;9:659764. doi: 10.3389/fchem.2021.659764 (PMC8341434; doi:10.3389/fchem.2021.659764)
Supplement: Supplementary file 1 [file DataSheet1.pdf]

Supplementary Materials

for

Identification of Potential Binding Sites of Sialic Acids on the RBD Domain of SARS-CoV-2 Spike Protein

Bingqian Li[1,2,#], Lin Wang[1,#], Huan Ge[3,#], Xianglei Zhang[1], Penxuan Ren[1], Yu Guo[4], Wuyan Chen[5], Jie Li[5], Wei Zhu[1], Wenzhang Chen[1], Lili Zhu[3,*], and Fang Bai[1,*]

[1]Shanghai Institute for Advanced Immunochemical Studies and School of Life Science and Technology, ShanghaiTech University, Shanghai 201210, China

[2]Department of Chemistry, Imperial College London, London SW7 2AZ, United Kingdom

[3]State Key Laboratory of Bioreactor Engineering, Shanghai Key Laboratory of New Drug Design, School of Pharmacy, East China University of Science and Technology, Shanghai 200237, China

[4]College of Pharmacy and State Key Laboratory of Medicinal Chemical Biology, Nankai University, Tianjin 300350, China

[5]National Center for Protein Science Shanghai, Shanghai 201210, China

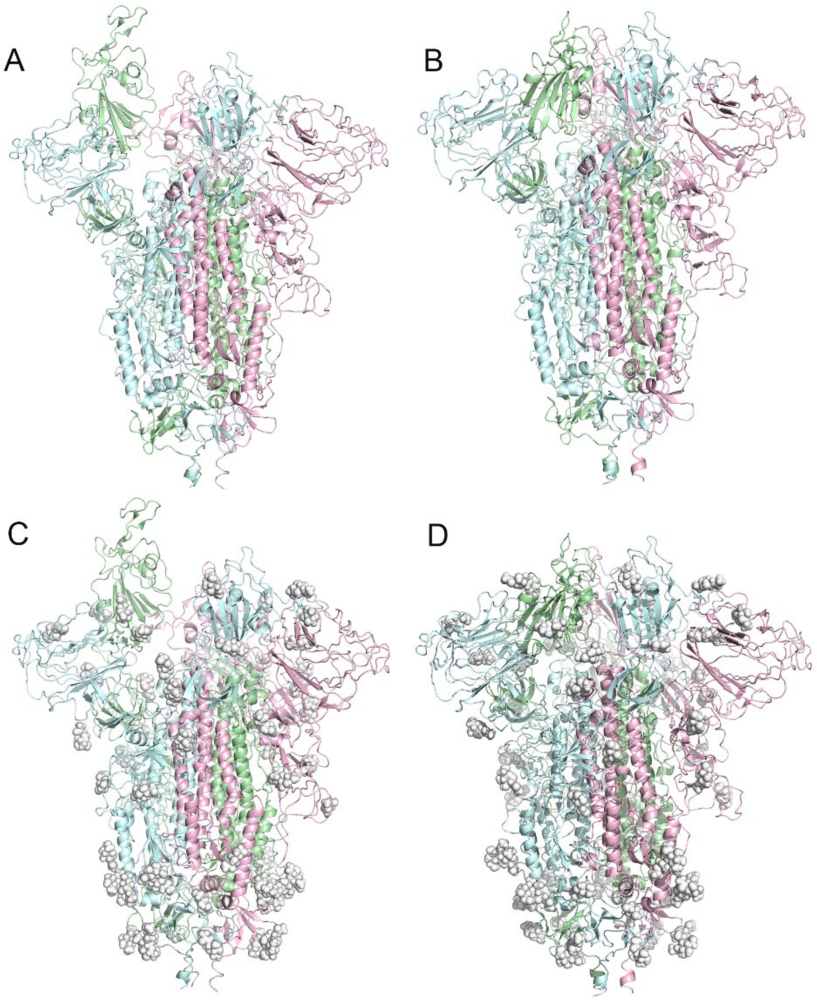

Figure S1. Models built for spike protein in different states. Four individual systems were built. (A) ‘up’ state spike protein without glycosylation; (B) ‘down’ state spike protein without glycosylation; (C) ‘up’ state spike protein with glycosylation; (D) ‘down’ state spike protein with glycosylation.

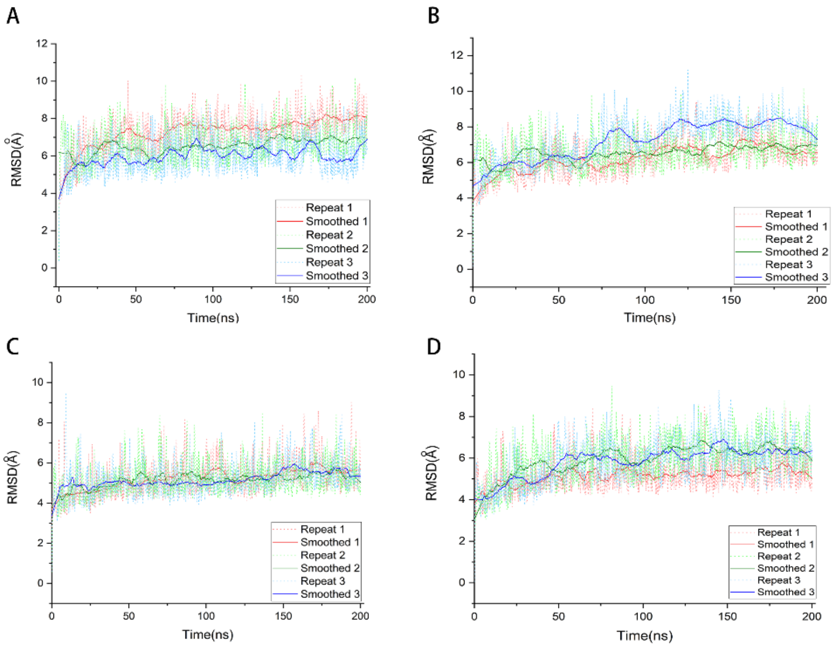

Figure S2. RMSD of trajectories. (A) RMSD of 'up' state without glycosylation. (B) RMSD of 'up' state with glycosylation. (C) RMSD of 'down' state without glycosylation. (D) RMSD of 'down' state with glycosylation. The raw data of RMSDs are shown in dot lines, and the fluctuations of RMSD are smoothed by using Savitzky-Golay method in OriginPro, version 2020 (OriginLab Corporation, Northampton, MA, USA), with the polynomial order as 1 and polynomial order as 50.

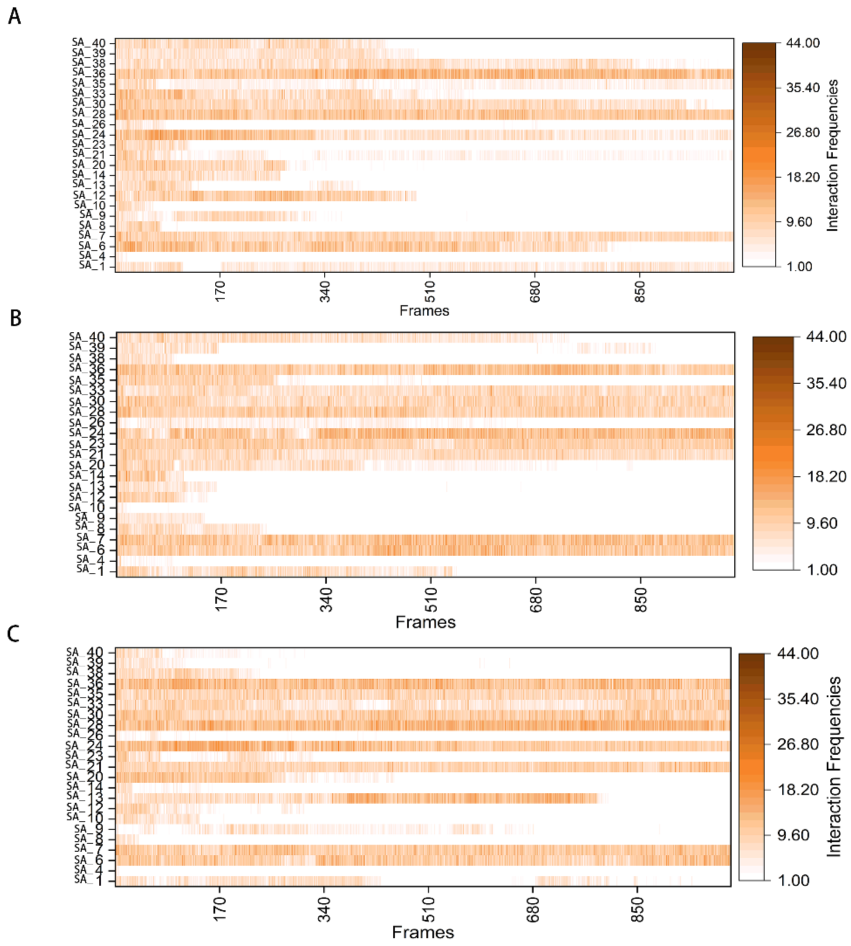

Figure S3. Heatmaps of 'down' state spike protein with glycosylation. The molecular dynamics simulation was repeated for three times. The heatmap shows the No. of ligand-protein interactions in each frame. The strength of color shows the number of interactions. The deeper the color, the more ligand-protein interactions in that frame. In all three repeats, the sialic acids at position SA_6, SA_7, SA_28, SA_30 and SA_36 are very stable. (A) Heatmap of 'down' state spike protein without glycosylation for repeat 1. (B) Heatmap of 'down' state spike protein without glycosylation for repeat 2. (C) Heatmap of 'down' state spike protein without glycosylation for repeat 3.

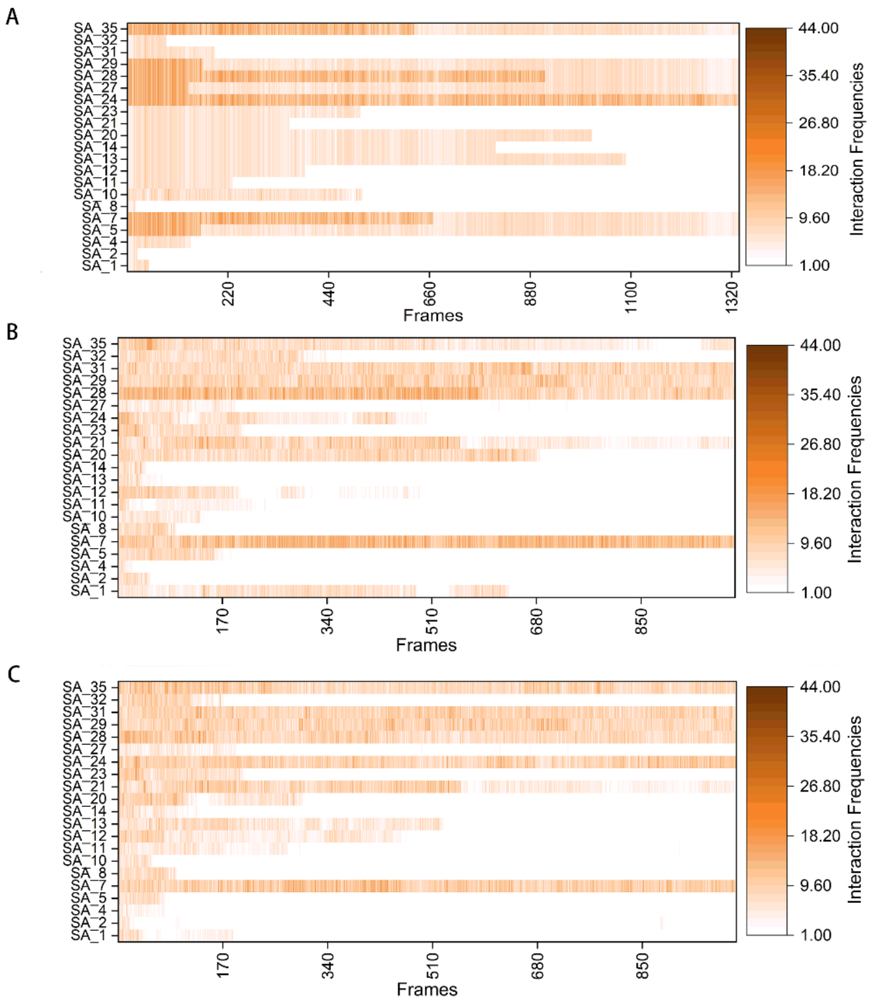

Figure S4. Heatmaps of 'down' state spike protein without glycosylation. The molecular dynamics simulation was repeated for three times. The heatmap shows the No. of ligand-protein interactions in each frame. The strength of color shows the number of interactions. The deeper the color, the more ligand-protein interactions in that frame. In all three repeats, the sialic acids at position SA_7, SA_24 and SA_28 are very stable. (A) Heatmap of 'down' state spike protein without glycosylation for repeat 1. (B) Heatmap of 'down' state spike protein without glycosylation for repeat 2. (C) Heatmap of 'down' state spike protein without glycosylation for repeat 3.

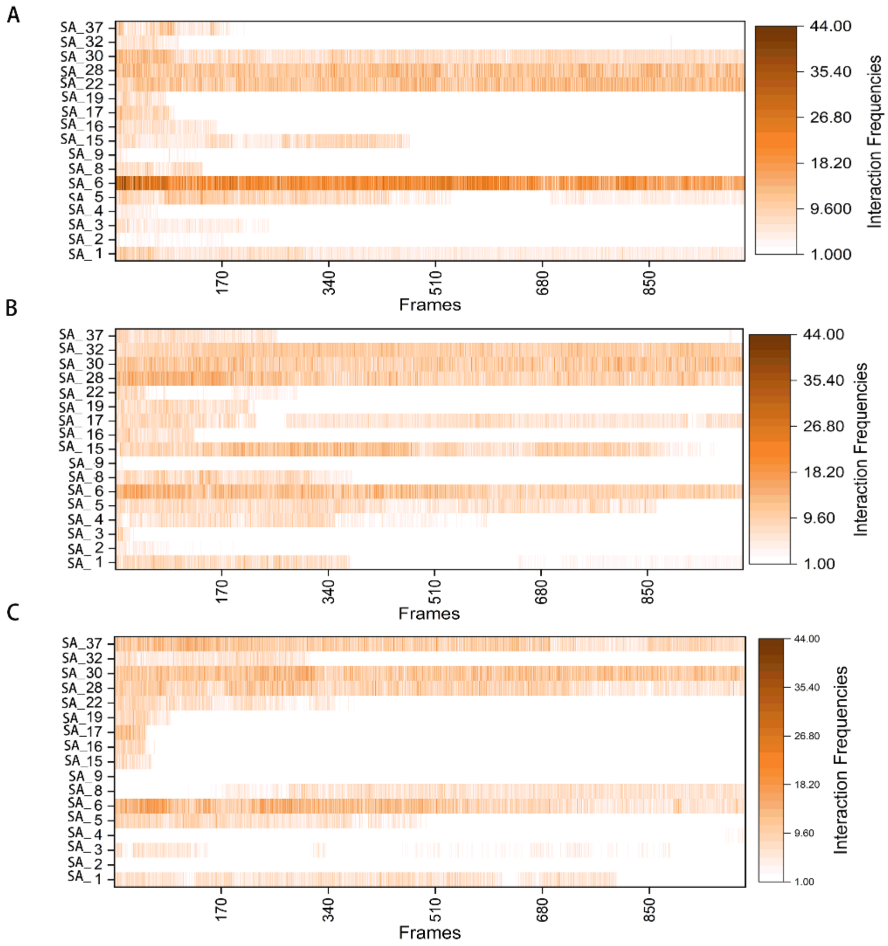

Figure S5. Heatmaps of 'up' state spike protein with glycosylation. The molecular dynamics simulation was repeated for three times. The heatmap shows the No. of ligand-protein interactions in each frame. The strength of color shows the number of interactions. The deeper the color, the more ligand-protein interactions in that frame. In all three repeats, the sialic acids at position SA_6, SA_28 and SA_30 are very stable. (A) Heatmap of 'up' state spike protein with glycosylation for repeat 1. (B) Heatmap of 'up' state spike protein with glycosylation for repeat 2. (C) Heatmap of 'up' state spike protein with glycosylation for repeat 3.

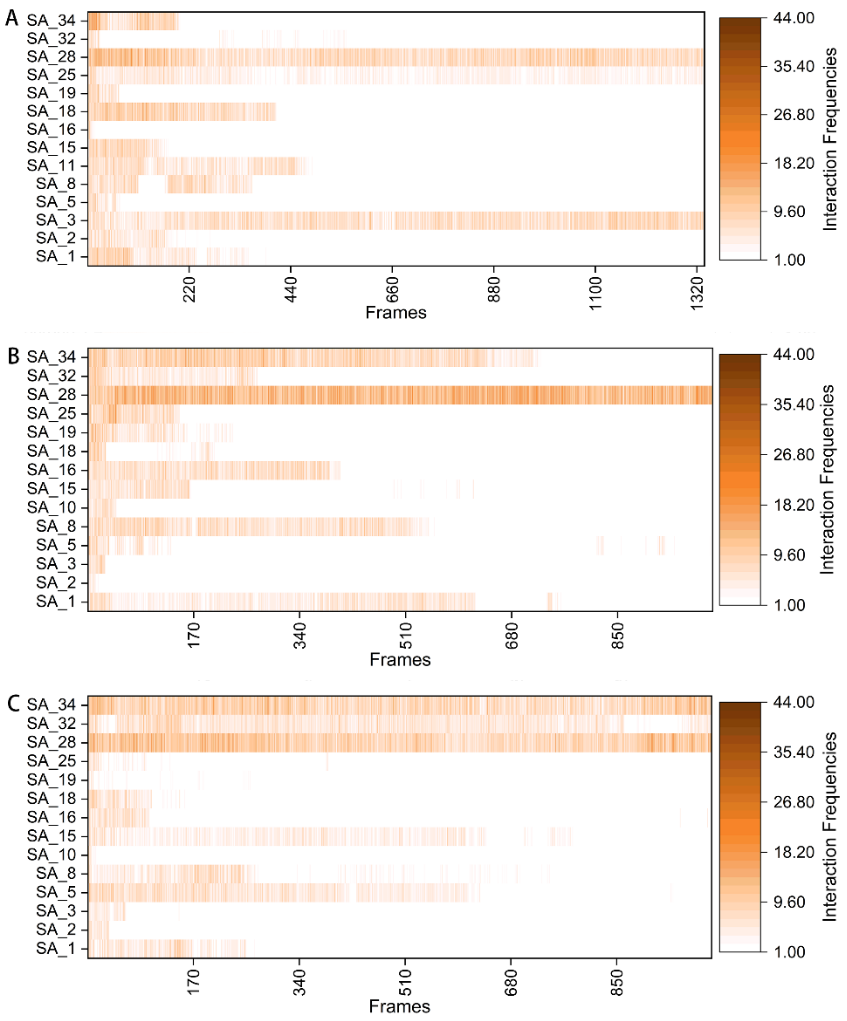

Figure S6. Heatmaps of 'up' state spike protein without glycosylation. The molecular dynamics simulation was repeated for three times. The heatmap shows the number of ligand-protein interactions in each frame which is represented by strength of color. The deeper the color, the more ligand-protein interactions in that frame. In all three repeats, the sialic acid at position 28 is very stable. (A) Heatmap of 'up' state spike protein without glycosylation for repeat 1. (B) Heatmap of 'up' state spike protein without glycosylation for repeat 2. (C) Heatmap of 'up' state spike protein without glycosylation for repeat 3.

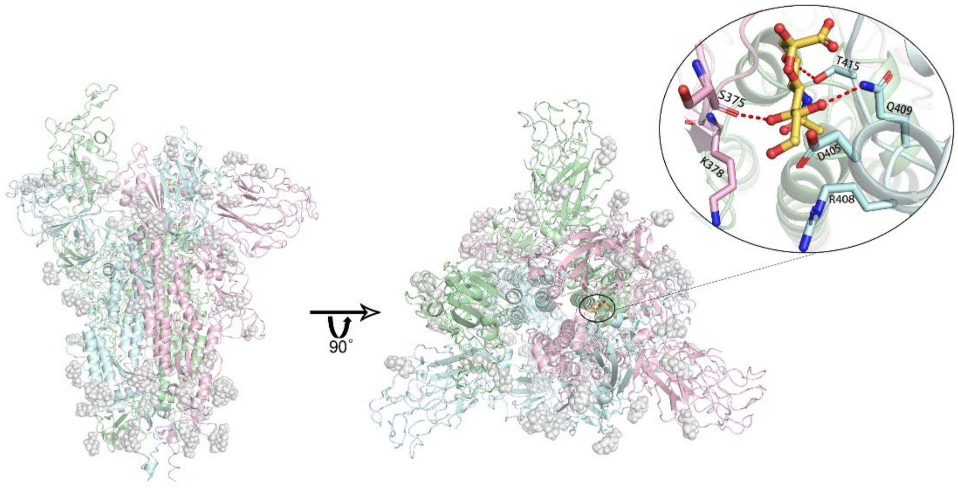

Figure S7. The predicted binding mode of sialic acid with glycosylated 'up' spike protein at the position of SA_6. Dash lines represent hydrogen or ionic bonds. White spheres are the glycans which are artificially modified on the protein.

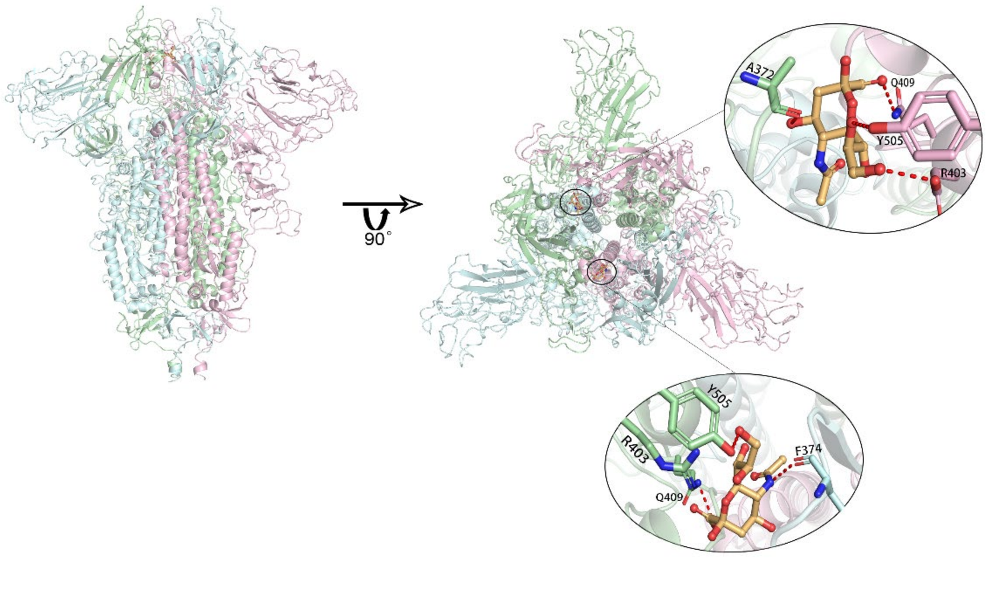

Figure S8. The predicted binding mode of sialic acid with unglycosylated 'down' spike protein at the position of SA_7 and SA_24. Dash lines represent hydrogen or ionic bonds

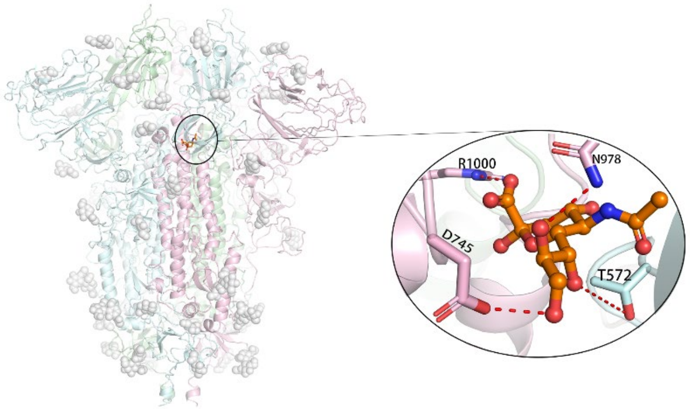

Figure S9. The computationally obtained binding mode of sialic acid in the position of SA_28.

Table S1. Reported single point mutations of Spike protein of SARS-CoV-2. This table lists the reported single point mutations on Spike proteins main sequences. (CNCB, 2021; CoVariant, 2021)

L5I, L5F, L18F, L18I, I68L, D80Y, S98F, S98C, S98Y, V143D, V143G, M153K, M153T, A222V, A262S, A262P, A262T, P272R, P272L, P272H, L276V, L276I, N439K, E484K, L452R, Y453F, S477N, S477T, S477I, N501Y, A570V, A570D, E583D, D614G, D614A, A626S, Q675H, Q677H, P681R, P681L, P681H, T716I, S982A, D1084E, D1118H, D1118Y, V1122L, …
